# Evaluation of Computerized Cognitive Training and Cognitive and Daily Function in Patients Living With HIV: A Meta-analysis

**DOI:** 10.1001/jamanetworkopen.2022.0970

**Published:** 2022-03-03

**Authors:** Jiaqi Wei, Jianhua Hou, Tingting Mu, Jun Sun, Shuang Li, Hao Wu, Bin Su, Tong Zhang

**Affiliations:** 1 Beijing Key Laboratory for HIV/AIDS Research, Clinical and Research Center for Infectious Diseases, Beijing Youan Hospital, Capital Medical University, Beijing, China; 2 Department of Social and Behavioural Sciences, City University of Hong Kong, Hong Kong Special Administrative Region, China; 3 Department of Radiology, Beijing Youan Hospital, Capital Medical University, Beijing, China

## Abstract

**Importance:**

In the era of antiretroviral therapy (ART), the incidence of HIV-associated neurocognitive disorder (HAND) has not yet been controlled. With the exception of ART, there is no beneficial pharmacologic treatment. However, some studies have reported that computerized cognitive training (CCT) programs may improve cognitive function among people living with HIV.

**Objective:**

To examine the association between CCT programs and 8 domains measuring cognitive function (7 domains) and daily function (1 domain) among people living with HIV.

**Data Sources:**

Records from the Cochrane Library, PsycINFO, PubMed, and Web of Science were searched from database inception to December 15, 2020. Supplementary searches to identify missing studies were conducted in Google Scholar using updated search terms from database inception to November 18, 2021.

**Study Selection:**

Studies that compared changes before and after a CCT intervention among people living with HIV were included. Search terms were a combination of words associated with HIV (eg, *people living with HIV*, *HIV*, and/or *AIDS*) and cognitive training (eg, *cognitive intervention*, *nonpharmacology intervention*, *computer game*, *video game*, *computerized training*, *cognitive exercise*, *cognitive stimulation*, and/or *cognitive enhancement*). Studies were included if they (1) used CCT as the primary intervention or combined CCT with other types of interventions; (2) used placebo, passive control conditions, traditional cognitive training, or single training tasks as control conditions; (3) reported changes between baseline and posttraining; (4) included participants 18 years or older; and (5) were randomized clinical trials (RCTs). Studies were excluded if they (1) were not associated with HIV, (2) were research protocols or feedback reports, (3) were case reports, or (4) did not report findings for domains of interest.

**Data Extraction and Synthesis:**

Two reviewers independently extracted data. This study followed the Preferred Reporting Items for Systematic Reviews and Meta-analyses (PRISMA) reporting guideline. Random-effects models were used to quantitatively synthesize the existing data.

**Main Outcomes and Measures:**

Primary outcomes were the meta-synthesized changes in each domain after CCT.

**Results:**

Among 1245 records identified, 1043 were screened after removal of duplicates. Of those, 1019 records were excluded based on titles and abstracts, and 24 full-text articles were assessed for eligibility. After exclusions, 12 eligible RCTs were selected for inclusion in the meta-analysis. These RCTs involved 596 total participants, with 320 individuals in the CCT group (mean age, 47.5-59.7 years; 0%-94% female; 8.3-14.2 years of education) and 276 individuals in the control group (mean age, 44.2-60.0 years; 19%-90% female; 9.0-14.9 years of education). The average HIV inhibition ratio (the proportion of participants who achieved virological suppression) ranged from 30% to 100%, and the CD4^+^ T-cell count ranged from 471 to 833 cells/μL. The time since training ranged from 3 to 24 weeks. After receipt of CCT, function significantly improved in 6 of the 8 domains: abstraction and executive function (standardized mean difference [SMD], 0.58; 95% CI, 0.26-0.91; *P* < .001), attention and working memory (SMD, 0.62; 95% CI, 0.33-0.91; *P* < .001), memory (SMD, 0.59; 95% CI, 0.20-0.97; *P* = .003), motor skills (SMD, 0.50; 95% CI, 0.24-0.77; *P* < .001), speed of information processing (SMD, 0.65; 95% CI, 0.37-0.94; *P* < .001), and daily function (SMD, 0.44; 95% CI, 0.02-0.86; *P* = .04). Sensory and perceptual skills (SMD, 0.06; 95% CI, −0.36 to 0.48; *P* = .78) and verbal and language skills (SMD, 0.46; 95% CI, −0.07 to 0.99; *P* = .09) did not significantly improve after CCT.

**Conclusions and Relevance:**

This meta-analysis of RCTs found that CCT programs were associated with improvements in cognitive and daily function among people living with HIV. Future studies are needed to design optimal specific training programs and use implementation science to enable the transformation of CCT from a scientific research tool to a real-world clinical intervention.

## Key Points

**Question:**

Are computerized cognitive training (CCT) programs associated with improvements in cognitive and daily function among people living with HIV?

**Findings:**

In this meta-analysis of 12 randomized clinical trials involving 596 participants living with HIV, CCT was associated with improved function in 6 of the 8 domains assessed, including abstraction and executive function, attention and working memory, memory, speed of information processing, motor skills, and daily function.

**Meaning:**

This meta-analysis found that CCT programs were associated with improvements in cognitive and daily function among people living with HIV.

## Teaser

This meta-analysis uses data from randomized clinical trials to assess the association of computerized cognitive training programs with cognitive and daily function among people living with HIV.

## Introduction

People living with HIV experience cognitive deterioration, also known as HIV-associated neurocognitive disorder (HAND), despite the widespread use of antiretroviral therapy (ART).^1,2,3,4^ Based on commonly used criteria,^5^ HAND is diagnosed by assessing 7 cognitive domains, including speed of information processing, sensory and perceptual (sensory/perceptual) skills, memory (learning and recall), attention and working memory (attention/working memory), motor skills, verbal and language (verbal/language) skills, and abstraction and executive function (abstraction/executive function).^6,7^ A diagnosis of HAND can have substantial consequences for an individual’s daily function.^8,9^ In addition, HAND has been associated with reduced life satisfaction^10,11,12^ and social isolation,^13^ producing public health and economic burdens worldwide.^14,15^ With the exception of ART, there is no specific pharmacologic treatment for HAND.^16,17^ However, studies have reported potential benefits from nonpharmacologic interventions that may ameliorate cognitive decline and reduce the odds of developing HAND among people living with HIV.^18,19^ These interventions have attracted the attention of many researchers. Chan et al^18^ focused on different types of cognitive and neurologic rehabilitation strategies among people living with HIV. Their results highlighted the importance of ART and suggested that nonpharmacologic strategies might improve cognitive function, either as stand-alone interventions or as part of a multidisciplinary approach.

Because of its safety, relevance, low cost, scalability, and convenience, computerized cognitive training (CCT) has been one of the most commonly used nonpharmacologic interventions.^20,21,22^ Computerized cognitive training programs aim to incorporate guided drills and practice for single or multiple cognitive domains through specific standardized procedures, differentiating CCT from other cognitive remediation approaches.^23^ The theoretical premise behind CCT is that it can stimulate neuroplasticity.^24^ From a neuropsychological perspective,^25^ CCT has the potential to shape brain structure and reorganize function among cognitively healthy older adults^26,27,28^ and individuals with Alzheimer disease,^29,30^ Parkinson disease,^31,32^ attention deficits,^33^ and acquired brain injury.^34^ Furthermore, physiological parameters, including brain metabolism^35^ and inflammatory,^36^ hormonal,^37^ and sleep-related factors,^38^ may also be transformed through brain plasticity.^25^ In addition, many studies involving people living with HIV have reported that CCT may reduce the risk of cognitive deterioration in several domains,^39^ such as working memory,^40,41,42,43^ speed of information processing,^40,44^ executive function and attention,^45,46,47^ and memory (learning and recall).^42^ However, the benefits reported in original articles using the same CCT program (Posit Science software) have been inconsistent. Pope et al^48^ found that this software could improve abstraction/executive function, whereas Fazeli et al^49^ reported that the software could not only enhance abstraction/executive function but also improve attention, working memory, and speed of information processing among people living with HIV.

Because of the mixed results reported in original studies, Vance et al^39^ investigated the findings in a systematic review. After identifying 13 items that fit their selection criteria, they found that most of the CCT programs were associated with improvements in cognitive function that translated into better daily function, improved mood, more substantial locus of control, and enhanced quality of life. Nevertheless, Vance et al^39^ did not calculate the effect size for each subdomain or perform sensitivity or moderator analyses. In addition, to our knowledge, no previous meta-analysis has confirmed the association of CCT with daily function and with each cognitive domain categorized by the Frascati criteria.^5^ The potential factors associated with CCT outcomes for each cognitive domain among people living with HIV have also not been confirmed.

Given these knowledge gaps, we conducted a meta-analysis to assess the associations of CCT programs with cognitive and daily function among people living with HIV. The present study aimed to (1) assess the extent of improvement in each domain after CCT among people living with HIV and (2) explore the consistency of the domain results among all of the potential factors. Furthermore, the study aimed to provide suggestions for future implementation of CCT interventions among people living with HIV.

## Methods

This study was registered in the International Prospective Register of Systematic Reviews (PROSPERO; registration No.: CRD42020210805). The study followed the Preferred Reporting Items for Systematic Reviews and Meta-analyses (PRISMA) reporting guideline for meta-analyses.^50^

### Search Strategy

We performed a record search of electronic databases, including the Cochrane Library, PsycINFO, PubMed, and Web of Science, with no limitations on publication type, from database inception to December 15, 2020. We also conducted a supplementary search using additional search terms from a previous meta-analysis.^51^ The search terms were a combination of words associated with HIV (eg, *people living with HIV*, *HIV*, and/or *AIDS*) and cognitive training (eg, *cognitive training*, *cognitive intervention*, *cognitive rehabilitation*, *nonpharmacology intervention*, *mnemonic training*, *processing speed training*, *working memory training*, *N-back training*, *attention training*, *reasoning training*, *computer game*, *video game*, *computerized training*, *computerized intervention*, *cognitive exercise*, *brain exercise*, *cognitive stimulation*, and/or *cognitive enhancement*). Additional searches to identify missing studies were also conducted from database inception to November 18, 2021, using Google Scholar and the reference lists of reviews and included studies. These supplementary searches did not identify any missing studies or studies published after the conclusion of the primary search (December 15, 2020). A full description of the initial and supplementary search strategies is available in eTable 1 and eTable 2 in the Supplement.

### Selection Criteria

**Figure 1.**
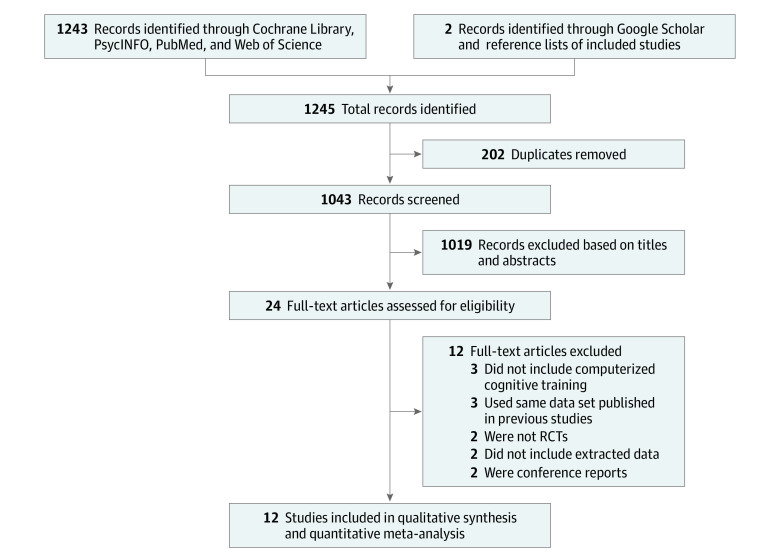
PRISMA Diagram RCT indicates randomized clinical trial.

Studies were eligible for inclusion if they (1) used CCT as the primary intervention or combined CCT with other types of interventions; (2) used a placebo, passive control conditions, traditional cognitive training, or single training tasks as control conditions; (3) reported changes between baseline and posttraining; (4) included participants 18 years or older and (5) were randomized clinical trials (RCTs). Studies were excluded if they (1) were not associated with HIV, (2) were research protocols or feedback reports, (3) were case reports, or (4) did not report findings for domains of interest. Duplicate studies were removed using EndNote X9 software (Clarivate), and 2 reviewers (J.W. and J.H.) separately selected search results based on titles and abstracts. Assessment of full-text articles to determine eligibility of the remaining studies was conducted by the same 2 reviewers. Disagreements about eligibility between reviewers were resolved through discussion with 2 investigators (B.S. and T.Z.). A detailed description of reasons for exclusion is available in eTable 3 in the Supplement, and the study selection process is shown in Figure 1.

### Data Extraction

Relevant data were independently extracted and cross-checked by 2 researchers (J.W. and J.H.) using an Excel spreadsheet (Microsoft Corporation). The summary statistics collected for each outcome included number of participants, means, and SDs. Training outcomes included daily function (1 domain) and cognitive tasks (7 domains, which included speed of information processing, sensory/perceptual skills, memory [learning and recall], attention/working memory, motor skills, verbal/language skills, and abstraction/executive function). The 7 cognitive domains were categorized using Frascati criteria^5^ and selected based on criteria from a previous study^52^ and work by Lezak et al.^53^ Details about the categorization process are shown in eTable 4 in the Supplement. Training dose was defined by the total number of training sessions, duration of each session, total training hours, training frequency, and time since training. Other information extracted from each RCT included the name of the first author, study location, year of publication, sample size, sex distribution, mean age and educational level of participants, current CD4^+^ T-cell counts, and current HIV inhibition ratio (the proportion of participants who achieved virological suppression).

### Statistical Analysis

Data analysis was performed using Comprehensive Meta-Analysis software, version 3 (Biostat, Inc), and the funnel plot was constructed using Review Manager software, version 5.4 (Cochrane Training). Because of the inherent heterogeneity across studies, we used random-effects models to estimate pooled effect sizes. Standardized mean differences (SMDs) were calculated as the mean change from pretraining to posttraining in the intervention group minus the mean change from pretraining to posttraining in the control group divided by the combined pretest SD (adjusted for bias). The inverse variance method was used to connect the SMDs of each study. We used *Q* tests (ie, χ^2^ tests) and the *I*^2^ statistic to assess the statistical and proportional significance of heterogeneity. We also used the Egger regression intercept test to estimate publication bias. The threshold for statistical significance was 2-tailed *P* < .05.

**Figure 2.**
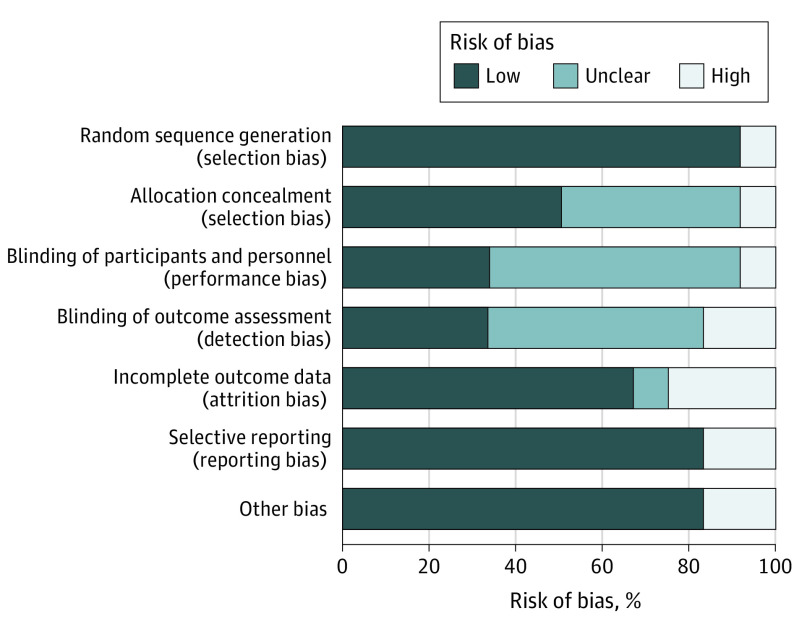
Risk of Bias

Study quality was evaluated using the Cochrane risk of bias tool, which divided risk of various biases into 3 grades: low, high, and unclear (Figure 2; eFigure in the Supplement). We used GRADEpro software, version 3.6 (McMaster University and Evidence Prime, Inc), to assess the methodological quality of included evidence (eTable 5 in the Supplement).

Sensitivity analyses were performed for significant results. The moderators included participant age (<18 years vs ≥18 years), proportion of women (<50% vs ≥50%), years of education (≤12 years vs >12 years), current CD4^+^ T-cell counts (<500 cells/μL vs ≥500 cells/μL), current HIV inhibition ratio (<100% vs 100%), total sessions (<22 vs ≥22), session duration (<60 minutes vs ≥60 minutes), session frequency (<3 sessions per week vs ≥3 sessions per week), total training time (<10 hours vs ≥10 hours), and time since training (<10 weeks vs ≥10 weeks). Study participants were also divided into those with normal vs impaired cognitive status.

## Results

### Search Results

Because the methods and results of supplementary research covered the initial research results, we used the flowchart from our supplementary research to describe study selection in this article (Figure 1). We included a total of 1245 records. After removing all duplicates (n = 202), 1043 records were screened. Of those, 1019 records were excluded based on titles and abstracts, and 24 full-text articles were assessed for eligibility. After exclusions, 12 eligible RCTs^40,41,42,44,45,46,47,48,49,54,55,56^ were selected for inclusion in the meta-analysis.

### Study Characteristics

Studies included data from the US,^40,41,44,46,47,48,49,54,55,56^ Uganda,^42^ and Italy,^45^ comprising 596 total participants (320 participants in the CCT group and 276 participants in the control group). Participant ages ranged from a mean of 47.5 years^45^ to 59.7 years^42^ in the CCT group and 44.2 years^46^ to 60.0 years^42^ in the control group. The proportion of women ranged from 0%^54^ to 94%^40^ in the CCT group and 19%^45^ to 90%^40^ in the control group. Years of education ranged from 8.3 years^42^ to 14.2 years^40^ in the CCT group and 9.0 years^45^ to 14.9 years^40^ in the control group. Only 9 studies^40,42,44,45,46,47,48,49,54^ reported etiologic data from CCT groups; in those studies, CD4^+^ T-cell counts ranged from 471 cells/μL^44^ to 833 cells/μL,^49^ and the HIV inhibition ratio ranged from 30%^44^ to 100%.^45^ Additional characteristics of the included RCTs and participants are shown in the Table.

**Table.** Study and Participant Characteristics

Source (location)	Sample										CCT intervention				Cognitive domain targeted by training	Control condition
	Description of participants	Sample size, No.			Age, mean, y		Female sex, %		Educational level, mean, y		Training	Sessions, No.	Session frequency per wk	Follow-up duration, wk		
		Total	CCT arm	Control arm	CCT arm	Control arm	CCT arm	Control arm	CCT arm	Control arm						
Chang et al,^40^ 2017 (US)	Adults with documented HIV seropositivity receiving ART for ≥6 mo	54	34	20	50.3	57.0	94	90	14.2	14.9	Adaptive Cogmed working memory training	25	3.5	4	Attention and working memory	Nonadaptive Cogmed working memory training
Cody et al,^41^ 2020 (US)	Older adults with or without HIV and without other physical or mental health disorders, hearing or vision problems, or history of brain trauma	33	17	16	56.0	55.6	35	31	12.5	12.6	Posit Science (BrainHQ.com); Target Tracker; tic’s	10	2	5	Speed of information processing; attention and working memory	Sham tDCS
Ezeamama et al,^42^ 2020 (Uganda)	Older adults (aged ≥50 y) with HIV and without other physical or mental health disorders	81	41	40	59.7	60.0	59	50	8.3	9.2	Captain’s Log MindPower Builder	2	2	5	Attention and working memory; memory	Standard of care
Fazeli et al,^49^ 2019 (US)	Older adults (aged ≥50 y) with HIV and without other physical or mental health disorders, hearing or vision problems, or history of brain trauma	33	17	16	56.0	55.6	35	31	12.5	12.6	Posit Science (BrainHQ.com); tDCS	10	NA	4	Speed of information processing	Sham tDCS
Livelli et al,^45^ 2015 (Italy)	Adults receiving care in division A infectious disease unit of Amedeo di Savoia Hospital	32	16	16	47.5	50.0	31	19	10.0	9.0	Combination of paper and pencil with computer-based exercises	36	NA	6	Attention and working memory; abstraction and executive function; memory; verbal and language skills	Standard of care
Ownby et al,^54^ 2017 (US)	Adults with HIV, self-reported cognitive difficulties, and cognitive impairment in 2 neuropsychological domains; no history of seizures or bipolar disorder; receiving psychotropic medications	11	6	5	50.3	52.8	0	40	12.0	10.2	GT Racing 2 (Gameloft); tDCS	6	3	3	Speed of information processing; abstraction and executive function	Sham tDCS
Pope et al,^48^ 2018 (US)	Older adults (aged ≥50 y) with HIV and without other physical or mental health disorders, hearing or vision problems, or history of brain trauma	30	15	15	55.3	53.7	33	40	12.5	12.3	Posit Science (BrainHQ.com); tDCS	10	4	NA	Speed of information processing	Sham tDCS
Towe et al,^46^ 2017 (US)	Adults (aged 18-65 y) with HIV receiving ART for >3 mo	21	11	10	51.3	44.2	27	20	14.1	12.6	Active cognitive training	12	2	10	Attention and working memory	Nonactive cognitive training
Towe et al,^47^ 2021 (US)	Adults (aged 18-64 y) with HIV infection, self-reported history of cocaine use lasting ≥1 y, receiving ART for >3 mo, English speaking, educational level >8 y, no other substantial neuromedical comorbidities or mental impairment, and not pregnant	58	29	29	49.0	48.3	17	38	12.1	11.9	Active cognitive training (Luminosity web-based cognitive games)	48	NA	10	Attention and working memory	Nonactive cognitive training
Vance et al,^44^ 2012 (US)	Adults (aged ≥40 y) with HIV for ≥1 y without other significant neuromedical comorbidities or mental impairment	46	22	24	50.1	52.9	23	29	13.3	13.1	Posit ScienceInSight computer program	NA	NA	5	Speed of information processing	No contact
Vance et al,^56^ 2021 (US)	Adults (aged ≥40 y) with HIV for ≥1 y living within 100 miles of research center and without other substantial neuromedical comorbidities or mental impairment	88	48	40	54.2	54.2	27	38	12.4	12.5	Individualized targeted cognitive training framework	NA	NA	12	Speed of information processing	No contact
Vance et al,^55^ 2021 (US)	Adults (aged ≥40 y) with HIV for ≥1 y living within 100 miles of research center and without other substantial neuromedical comorbidities or mental impairment	109	64	45	53.4	53.8	28	33	12.1	12.4	Individualized targeted cognitive training framework	NA	NA	12	Speed of information processing	No contact

Abbreviations: ART, antiretroviral therapy; CCT, computerized cognitive training; NA, not applicable; tDCS, transcranial direct current stimulation.

The total number of training sessions ranged from 6^54^ to 48,^47^ and session duration ranged from 20 minutes^54^ to 90 minutes.^56^ Training frequency ranged from 2 sessions per week^41,42,46^ to 4 sessions per week,^48^ total number of training hours ranged from 1.7^54^ to 20.0,^55,56^ and time since training ranged from 3 weeks^54^ to 24 weeks.^45^

Although all 12 studies^40,41,42,44,45,46,47,48,49,54,55,56^ used CCT programs, the intervention tools were different. Cody et al,^41^ Fazeli et al,^49^ and Pope et al^48^ used the same cognitive training program (BrainHQ.com; Posit Science) along with transcranial direct current stimulation (tDCS). Ownby et al^54^ also used tDCS combined with a video game (GT Racing 2; Gameloft). Towe et al^46,47^ used an active cognitive training tool (Lumosity web-based cognitive games; Lumos Labs, Inc). Chang et al^40^ used an adaptive working memory training platform (Cogmed; Neural Assembly), Ezeamama et al^42^ used computerized cognitive rehabilitation therapy software (Captain’s Log MindPower Builder; Brain Train, Inc), and Livelli et al^45^ combined paper and pencil and computer-based exercises. Vance et al^44,55,56^ used a computer program (InSight; Posit Science) in their 2012 study^44^ and an individualized targeted cognitive training framework in their 2021 studies.^55,56^

The control conditions were divided into 3 types: placebo (6 studies^41,42,45,48,49,54^ used sham tDCS or standard of care), no contact (3 studies^44,55,56^), and other (2 studies^46,47^ used nonactive cognitive training and 1 study^40^ used nonadaptive working memory training [Cogmed; Neural Assembly]). Six studies^44,45,48,49,54,56^ reported outcomes for the abstraction/executive function domain, 9 studies^40,41,44,45,46,47,49,54,56^ for the attention/working memory domain, 5 studies^41,42,49,54,56^ for the memory domain, 5 studies^42,45,49,54,56^ for the motor skills domain, 5 studies^41,42,44,47,54^ for the speed of information processing domain, 3 studies^44,45,55^ for the daily function domain, 1 study^56^ for the sensory/perceptual skills domain, and 4 studies^42,45,47,49^ for the verbal/language skills domain.

### Meta-analysis of Cognitive and Daily Function Domains

**Figure 3.**
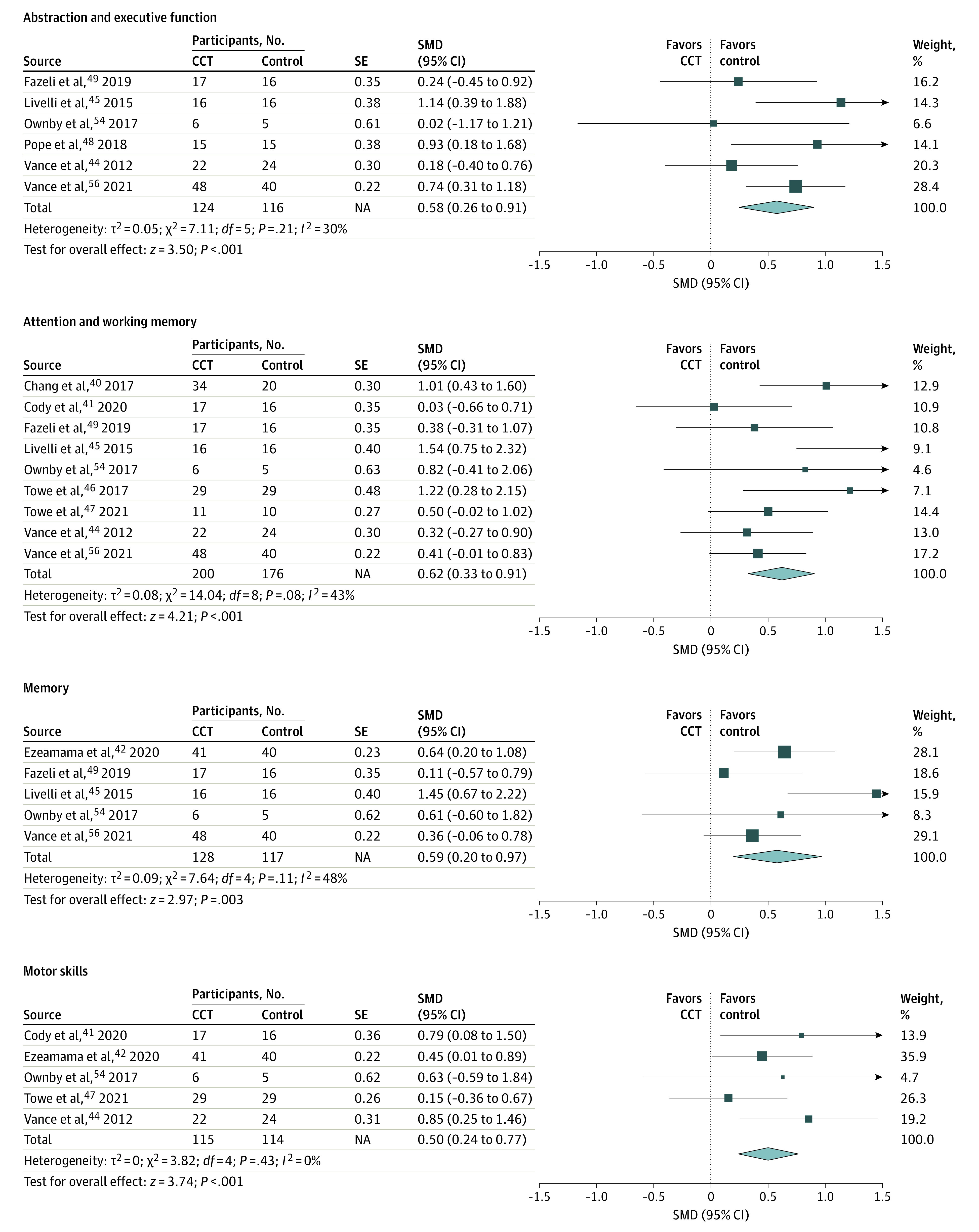
Pooled Effects of Abstraction and Executive Function, Attention and Working Memory, Memory, and Motor Skills CCT indicates computerized cognitive training; NA, not applicable; and SMD, standardized mean difference.

**Figure 4.**
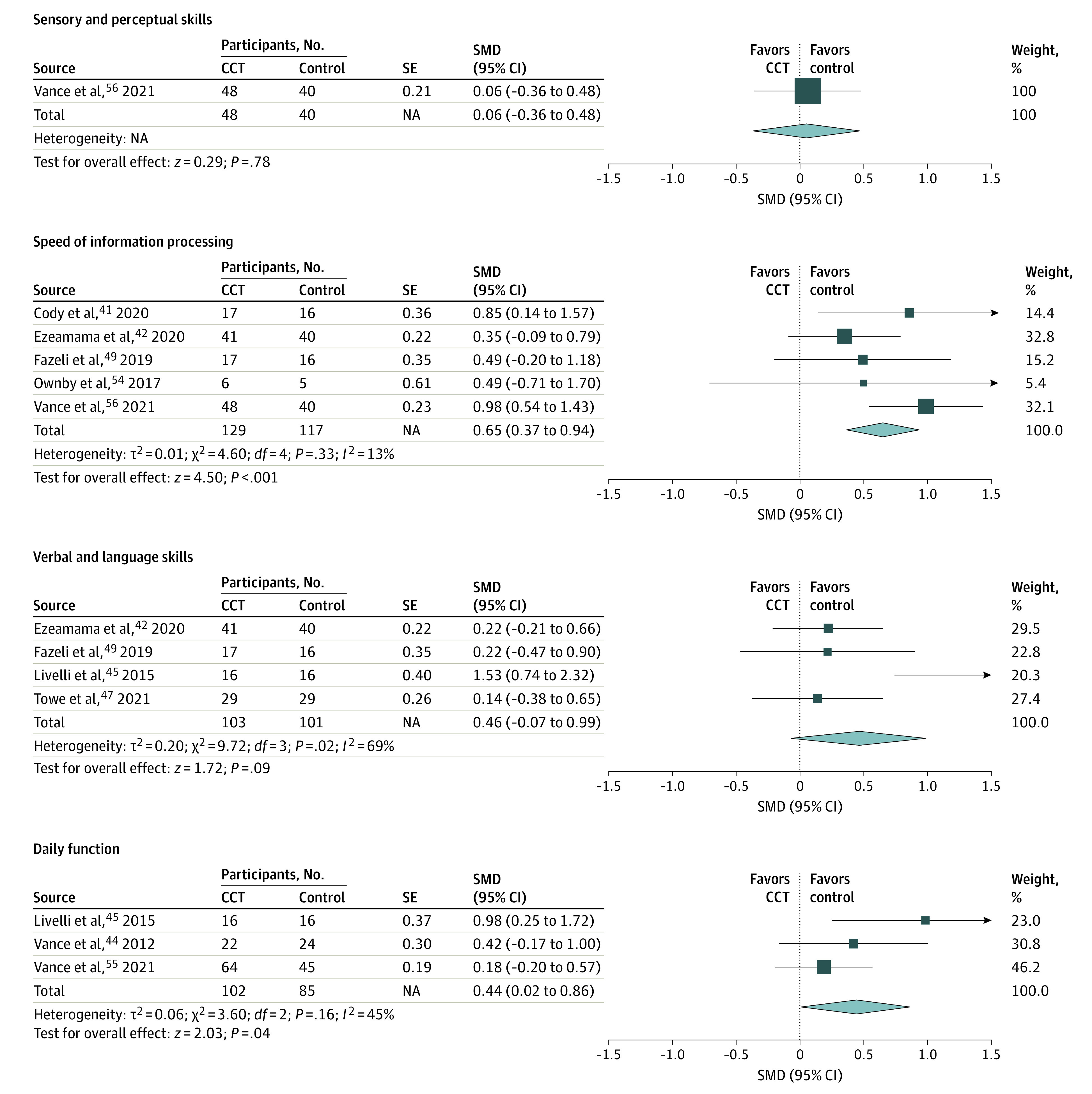
Pooled Effects of Sensory and Perceptual Skills, Information Processing Speed, Verbal and Language Skills, and Daily Function CCT indicates computerized cognitive training; NA, not applicable; and SMD, standardized mean difference.

Computerized cognitive training was significantly associated with improvements in 6 of the 8 domains: abstraction/executive function, attention/working memory, memory, motor skills, speed of information processing, and daily function. The detailed results of the meta-analysis for each domain are shown in Figure 3 and Figure 4.

#### Abstraction/Executive Function

Among 6 studies^44,45,48,49,54,56^ including 240 participants, the SMD for abstraction/executive function was 0.58 (95% CI, 0.26-0.91; *P* < .001). No significant heterogeneity (*Q* = 7.11; *I*^2^ = 30%; *P* = .21) or publication bias (intercept, −0.70; 95% CI, −5.92 to 4.52; *P* = .73) was found.

#### Attention/Working Memory

Among 9 studies^40,41,44,45,46,47,49,54,56^ involving 376 participants, the SMD for attention/working memory was 0.62 (95% CI, 0.33-0.91; *P* < .001). Moderate heterogeneity was detected (*Q* = 14.04; *I*^2^ = 43%; *P* = .08). No significant publication bias was found (intercept, 1.98; 95% CI, −1.69 to 5.65; *P* = .24).

#### Memory

Among 5 studies^41,42,49,54,56^ comprising 245 participants, the SMD for memory was 0.59 (95% CI, 0.20-0.97; *P* < .001). Moderate heterogeneity was detected (*Q* = 7.64; *I*^2^ = 48%; *P* = .11). No significant publication bias was found (intercept, 1.13; 95% CI, −5.29 to 7.55; *P* = .61).

#### Motor Skills

Among 5 studies^42,45,49,54,56^ involving 229 participants, the SMD for motor skills was 0.50 (95% CI, 0.24-0.77; *P* < .001). No significant heterogeneity (*Q* = 3.82; *I*^2^ = 0%; *P* = .43) or publication bias (intercept, 1.29; 95% CI, −3.71 to 6.30; *P* = .47) was found.

#### Speed of Information Processing

Among studies^41,42,44,47,54^ including 246 participants, the SMD for speed of information processing was 0.65 (95% CI, 0.37-0.94; *P* < .001). No significant heterogeneity (*Q* = 4.60; *I*^2^ = 13%; *P* = .33) or publication bias (intercept, −0.22; 95% CI, −5.72 to 5.29; *P* = .91) was detected.

#### Daily Function

Among 3 studies^44,45,55^ comprising 187 participants, the SMD for daily function was 0.44 (95% CI, 0.02-0.86; *P* = .04). No significant heterogeneity (*Q* = 3.60; *I*^2^ = 45%; *P* = .16) or publication bias (intercept, 3.87; 95% CI, −12.58 to 20.31; *P* = .21) was detected.

#### Sensory/Perceptual Skills

One study^56^ comprising 88 participants examined sensory/perceptual skills. The SMD was 0.06 (95% CI, −0.36 to 0.48; *P* = .78).

#### Verbal/Language Skills

Among 4 studies^42,45,47,49^ involving 204 participants the SMD for verbal/language skills was 0.46 (95% CI, −0.07 to 0.99; *P* = .09). Moderate heterogeneity was detected (*Q* = 9.72; *I*^2^ = 69%; *P* = .02). However, no significant publication bias was found (intercept, 5.04; 95% CI, −9.55 to 19.63; *P* = .28).

### Sensitivity Analysis

We conducted sensitivity analyses among all of the factors extracted from RCTs for each domain that had statistically significant results in the meta-analysis. Only 5 factors produced substantial changes in outcomes: age (memory domain: *Q* = 0.75; *P* = .02), session hours (attention/working memory domain: *Q* = 9.41; *P* < .001), time since training (abstraction/executive function domain: *Q* = 5.33; *P* = .02), CD4^+^ T-cell count (attention/working memory domain: *Q* = 4.78; *P* = .03), and HIV inhibition ratio (attention/working memory domain: *Q* = 4.52; *P* = .03; memory domain: *Q* = 4.76; *P* = .03). Detailed results from sensitivity analyses are available in eTable 6 in the Supplement.

### Study Quality and Risk of Bias

Eleven studies^40,41,42,44,45,46,47,48,49,54,55^ had moderate-quality evidence, and 1 study^56^ had very low-quality evidence (eTable 5 in the Supplement). Six domains (abstraction/executive function, attention/working memory, memory, motor skills, speed of information processing, and daily function) had moderate-quality evidence, for which the main reasons were moderate to high risk of bias, small samples, inclusion of few studies, and large 95% CIs. Two domains (sensory/perceptual and verbal/language skills) had very low-quality evidence.

Most studies had a low risk of bias for random sequence generation (11 studies^40,41,44,45,46,47,48,49,54,55,56^), selective reporting (10 studies^41,42,44,45,46,48,49,54,55,56^), incomplete outcome data (8 studies^40,42,45,46,47,49,55,56^), and other types of bias (10 studies^40,42,44,45,47,48,49,54,55,56^) (Figure 2; eFigure in the Supplement). Six studies^41,47,48,54,55,56^ (50.0%) reported allocation concealment as a risk of bias. Eight studies^40,42,44,45,46,47,55,56^ (66.7%) did not blind those who implemented or assessed the interventions and/or those who reported relevant information.

## Discussion

To our knowledge, this meta-analysis of 12 RCTs^40,41,42,44,45,46,47,48,49,54,55,56^ is the first to assess the association of CCT programs with cognitive and daily function among people living with HIV. Significant improvement was found in daily function and most cognitive domains, with the exception of sensory/perceptual and verbal/language skills, after CCT. Our results revealed no publication bias. The findings of the sensitivity analyses revealed that all pooled effects, with the exception of the memory and verbal/language skills domains, were statistically significant.

Moderate effect sizes for the speed of information processing and the attention/working memory domains suggested the potential for future improvement. Consistent with findings of previous meta-analyses of CCT interventions,^23,37,51,57^ these 2 domains were also more likely to improve after CCT among individuals with mild cognitive impairment^23,37^ and older adults with healthy cognitive status.^51,57^ Because improvements after cognitive training typically reflect training content,^57,58^ this result may be a result of sufficient training on these 2 subdomains within studies. Previous studies have also found that functional connectivity in the frontal-parietal brain network, mainly involved in the speed of information processing and the attention/working memory domains, increases after training.^59,60,61^ With regard to the sensory/perceptual and verbal/language skills domains, the null effect may be a result of the small number of studies and the presence of measurement bias because associations between these 2 domains and CCT have been found in other populations.^51,57^ Future studies may consider dedicating more time to targeting these 2 domains. Multiple studies involving cognitively healthy adults^57^ and individuals with Alzheimer disease^62^ have reported that training benefits extended beyond the cognitive domains assessed after training to include other cognitive domains, and these benefits have transferred to daily function, psychological health, and other higher-order competencies.^63,64^ We also found an association between CCT and daily function among people living with HIV. Despite the limitations of these training tools,^63^ preliminary data suggest that CCT can, in principle, improve a broader range of essential functions, including cognitive and daily function.^57^

With regard to the sensitivity analysis, fewer results were statistically significant, and analysis of the same factor applied to different domains yielded substantially different results. We did not find any statistical difference in the characteristics of study participants. Our results partially replicated those reported in a meta-analysis of CCT programs among patients with Alzheimer disease.^65^ Karssemeijer et al^65^ found only a slight difference in the benefits of CCT among older adults with mild cognitive impairment vs dementia. Hill et al^23^ reported that CCT was beneficial for global cognition in the memory and learning domains among people with mild cognitive impairment. In comparison, the evidence for benefit among people with dementia was weak.^23^ Possible reasons might include (1) the cognitive enhancement mechanism after CCT may have differed between the 2 groups, and (2) differences in the training methods and measurement tools used may have produced different results.

Regarding CCT dose, our analyses suggested that better results occurred after longer CCT sessions (ie, >60 minutes), possibly because synaptic plasticity is more likely to occur after 30 to 60 minutes of stimulation.^57,66^ A meta-analysis involving studies of patients with Alzheimer disease reached similar conclusions.^57^ Therefore, longer training sessions might be the recommended approach for people living with HIV. In contrast, many commercial products designed for at-home training use protocols consisting of shorter sessions, which may not be adequate. The findings of the current meta-analysis also provided a better understanding of viral load and current CD4^+^ T-cell counts among people living with HIV. Those with undetectable viral loads and current CD4^+^ T-cell counts of more than 500 cells/μL performed better than those with detectable viral loads and lower CD4^+^ T-cell counts, particularly in the attention/working memory domain. A previous study also found that people living with HIV who had current CD4^+^ T-cell counts lower than 500 cells/μL were more likely to have HAND.^4^ The pathogenesis of HAND may explain this phenomenon. After entering the central nervous system, HIV can stimulate chronic neuroinflammation, which interacts with viral proteins and produces cognitive impairment.^67^ Therefore, people with lower viral loads and higher CD4^+^ T-cell counts, which indicate better immune status, may experience more benefit from CCT.

Only 50% of the included RCTs^41,47,48,54,55,56^ reported allocation concealment as a risk of bias, producing inflated effect sizes and potential selection bias.^68^ Eight of 12 RCTs^40,42,44,45,46,47,55,56^ (66.7%) did not blind those who implemented or assessed the interventions and/or those who reported relevant information. These limitations may have created implementation and assessor biases, producing increases in false-positive results. With regard to the quality of the evidence, 2 domains (sensory/perceptual and verbal/language skills) were considered to have very low-quality evidence, and the remaining 6 domains (abstraction/executive function, attention/working memory, memory, motor skills, speed of information processing, and daily function) had moderate-quality evidence, for which the main reasons were moderate to high risk of bias, small samples, inclusion of few RCTs, and wide 95% CIs. This result suggested that our results need to be further explored. We found no significant publication bias across outcomes, suggesting our outcomes were statistically significant.

### Limitations

This study has limitations. First, the number of studies included in the meta-analysis was small, producing heterogeneity and low evidence quality. Therefore, the optimal intervention design for eliciting beneficial outcomes remains unclear. Second, the included RCTs also lacked measurement of blood, cerebrospinal fluid, and brain imaging biomarkers after CCT. Including such measurements could have helped to clarify the potential mechanisms of the benefits observed after CCT. Third, the RCTs did not measure concurrent treatment (eg, ART, tDCS, physical exercise, or mindfulness) during the CCT intervention. Analysis of synergistic factors and outcomes can help physicians develop more beneficial plans for patients.

## Conclusions

The findings of this meta-analysis of pooled data from RCTs suggested that CCT programs were associated with significant improvements in 6 cognitive and daily function domains (including abstraction/executive function, attention/working memory, memory, motor skills, speed of information processing, and daily function) among people living with HIV. Future studies are needed to clarify whether there is a difference in training benefits between CCT programs and to examine the synergistic factors and outcomes of different auxiliary interventions (eg, tDCS or exercise). In addition, more studies are needed to confirm the impact of potential factors and to assess training protocols among a large population of individuals living with HIV who are at risk of developing HAND. Studies in the field of implementation science are especially needed to address the challenge of removing barriers and bringing CCT from scientific research into clinical practice and implementing CCT programs in the real world.

## Notes

Supplement. **eTable 1.** Full Search Strategy **eTable 2.** Supplementary Search Strategy **eTable 3.** Reasons for Exclusion in Full-text Assessment **eTable 4.** Categorization of Outcome Measures **eTable 5.** Study Quality of Evidence According to GRADE Guidelines **eTable 6.** Full Results of Sensitivity Analyses **eFigure.** Risk of Bias Summary
