# Supplementary material for: Evaluation of Computerized Cognitive Training and Cognitive and Daily Function in Patients Living With HIV: A Meta-analysis
Source: JAMA Netw Open. 2022 Mar 3;5(3):e220970. doi: 10.1001/jamanetworkopen.2022.0970 (PMC8895263; doi:10.1001/jamanetworkopen.2022.0970)
Supplement: Supplement. — eTable 1. Full Search Strategy eTable 2. Supplementary Search Strategy eTable 3. Reasons for Exclusion in Full-text Assessment eTable 4. Categorization of Outcome Measures eTable 5. Study Quality of Evidence According to GRADE Guidelines eTable 6. Full Results of Sensitivity Analyses eFigure. Risk of Bias Summary [file jamanetwopen-e220970-s001.pdf]

Supplementary Online Content

Wei J, Hou J, Mu T, et al. Evaluation of computerized cognitive training and cognitive and daily function in patients living with HIV: a meta-analysis. *JAMA Netw Open*. 2022;5(3):e220970. doi:10.1001/jamanetworkopen.2022.0970

eTable 1. Full Search Strategy

eTable 2. Supplementary Search Strategy

eTable 3. Reasons for Exclusion in Full-text Assessment

eTable 4. Categorization of Outcome Measures

eTable 5. Study Quality of Evidence According to GRADE Guidelines

eTable 6. Full Results of Sensitivity Analyses

eFigure. Risk of Bias Summary

This supplementary material has been provided by the authors to give readers additional information about their work.

© 2022 Wei J et al. *JAMA Network Open*.

eTable 1. Full Search Strategy

Database	Time	Strategy	Outcome
Web of science	2020.12.15	("cognitive training" OR "cognitive intervention" OR "cognitive rehabilitation" OR "non-pharmacology intervention" OR "mnemonic training" OR "processing speed training" OR "working memory training" OR "N-back training") AND ("PLWH" OR "HIV" OR "AIDS")	271
PubMed	2020.12.15	("cognitive training" OR "cognitive intervention" OR "cognitive rehabilitation" OR "non-pharmacology intervention" OR "mnemonic training" OR "processing speed training" OR "working memory training" OR "N-back training") AND ("PLWH" OR "HIV" OR "AIDS")	92
PsycINFO	2020.12.15	(“cognitive training” OR “cognitive intervention” OR “cognitive rehabilitation” OR “non-pharmacology intervention” OR “mnemonic training” OR “processing speed training” OR “working memory training” OR “N-back training”) AND (“PLWH” OR “HIV” OR “AIDS”)	140
Cochrane	2020.12.15	“cognitive training” OR “cognitive intervention” OR “cognitive rehabilitation” OR “non-pharmacology intervention” OR “mnemonic training” OR “processing speed training” OR “working memory training” OR “N-back training” in Title Abstract Keyword AND “PLWH” OR “HIV” OR “AIDS” in Title Abstract Keyword	501

© 2022 Wei J et al. *JAMA Network Open*.

eTable 2. Supplementary Search Strategy

Database	Time	Strategy	Outcome
Web of science	2021.11.18	("cognitive training" OR "cognitive intervention" OR "cognitive rehabilitation" OR "non-pharmacology intervention" OR "mnemonic training" OR "processing speed training" OR "working memory training" OR "N-back training" OR "attention training" OR "reasoning training" OR "computer game" OR "video game" OR "computerized training" OR "computerized intervention" OR "cognitive exercise" OR "brain exercise" OR "cognitive stimulation" OR "cognitive enhancement") AND ("PLWH" OR "HIV" OR "AIDS")	512
PubMed	2021.11.18	("cognitive training" OR "cognitive intervention" OR "cognitive rehabilitation" OR "non-pharmacology intervention" OR "mnemonic training" OR "processing speed training" OR "working memory training" OR "N-back training" OR "attention training" OR "reasoning training" OR "computer game" OR "video game" OR "computerized training" OR "computerized intervention" OR "cognitive exercise" OR "brain exercise" OR "cognitive stimulation" OR "cognitive enhancement") AND ("PLWH" OR "HIV" OR "AIDS")	153
PsycINFO	2021.11.18	TX ("cognitive training" OR "cognitive intervention" OR "cognitive rehabilitation" OR "non-pharmacology intervention" OR "mnemonic training" OR "processing speed training" OR "working memory training" OR "N-back training" OR "attention training" OR "reasoning training" OR "computer game" OR "video game" OR "computerized training" OR "computerized intervention" OR "cognitive exercise" OR "brain exercise" OR "cognitive stimulation" OR "cognitive enhancement") AND TX ("PLWH" OR "HIV" OR "AIDS")	199
Cochrane	2021.11.18	“cognitive training” OR “cognitive intervention” OR “cognitive rehabilitation” OR “non-pharmacology intervention” OR “mnemonic training” OR “processing speed training” OR “working memory training” OR “N-back training” in Title Abstract Keyword AND “PLWH” OR “HIV” OR “AIDS” in Title Abstract Keyword<br><br>AND	379

© 2022 Wei J et al. *JAMA Network Open*.

		"attention training" OR "reasoning training" OR "computer game" OR "video game" OR "computerized training" OR "computerized intervention" OR "cognitive exercise" OR "brain exercise" OR "cognitive stimulation" OR "cognitive enhancement" in Title Abstract Keyword AND “PLWH” OR “HIV” OR “AIDS” in Title Abstract Keyword	
Google Scholar	2021.11.18	HIV AND cognitive training	314000

© 2022 Wei J et al. *JAMA Network Open*.

eTable 3. Reasons for Exclusion in Full-text Assessment

No.	Full text being excluded	Reasons
1	Avci G, Woods SP, Verduzco M, Sheppard DP, Sumowski JF, Chiaravalloti ND, DeLuca J; HIV Neurobehavioral Research Program (HNRP) Group. Effect of Retrieval Practice on Short-Term and Long-Term Retention in HIV+ Individuals. *J Int Neuropsychol Soc*. 2017 Mar;23(3):214-222.	Not having computerized cognitive training
2	Bai F, Allegrini M, Falcinella C, et al. Efficacy of a computerised cognitive rehabilitation training in improving HIV-associated neurocognitive disorders. *Journal of the international AIDS society*. 2018, 21.	Being a conference report
3	Bailey M D, Lohaugen G, Hanna-Pladdy B, et al. Adaptive working memory training on cognitive performance in HIV-1 patients. *Journal of neuroimmune pharmacology*. 2019, 14(2): 352-352.	Being a conference report
4	Becker JT, Dew MA, Aizenstein HJ, Lopez OL, Morrow L, Saxton J, Tárraga L. A pilot study of the effects of internet-based cognitive stimulation on neuropsychological function in HIV disease. *Disabil Rehabil.* 2012;34(21):1848-52.	Not having extracted data
5	Casaletto KB, Moore DJ, Woods SP, Umlauf A, Scott JC, Heaton RK. Abbreviated Goal Management Training Shows Preliminary Evidence as a Neurorehabilitation Tool for HIV-associated Neurocognitive Disorders among Substance Users. *Clin Neuropsychol*. 2016;30(1):107-30.	Not having computerized cognitive training
	Chan, T., Marta, M., Hawkins, C., & Rackstraw, S. (2020). Cognitive and Neurologic Rehabilitation Strategies for Central Nervous System HIV Infection. Current HIV/AIDS reports, 17(5), 514–521.	Being a review
6	Chang L, Løhaugen GC, Douet V, Miller EN, Skranes J, Ernst T. Neural correlates of working memory training in HIV patients: study protocol for a randomized controlled trial. *Trials.* 2016 Feb 2; 17:62.	Having the same dataset published in previous studies
7	Cody SL, Fazeli PL, Vance DE. Feasibility of a Home-Based Speed of Processing Training Program in Middle-Aged and Older Adults With HIV. *J Neurosci Nurs*. 2015 Aug;47(4):247-54.	Not having computerized cognitive training
8	Frain JA, Chen L. Examining the effectiveness of a cognitive intervention to improve cognitive function in a population of older adults living with HIV: a pilot study. *Ther Adv Infect Dis*. 2018 Jan;5(1):19-28.	Not having extracted data

© 2022 Wei J et al. *JAMA Network Open*.

9	Ownby RL, Acevedo A. A pilot study of cognitive training with and without transcranial direct current stimulation to improve cognition in older persons with HIV-related cognitive impairment. *Neuropsychiatr Dis Treat.* 2016 Oct 25;12:2745-2754.	Not being RCT design
	van der Heijden, I., Abrahams, N., & Sinclair, D. (2017). Psychosocial group interventions to improve psychological well-being in adults living with HIV. The Cochrane database of systematic reviews, 3(3), CD010806.	Being a review
	Vance, D. E., Fazeli, P. L., Grant, J. S., Slater, L. Z., & Raper, J. L. (2013). The role of neuroplasticity and cognitive reserve in aging with HIV: recommendations for cognitive protection and rehabilitation. The Journal of neuroscience nursing: journal of the American Association of Neuroscience Nurses, 45(5), 306–316.	Being a review
10	Vance DE, Gakumo CA, Childs GD, Enah C, Fazeli PL. Feedback on a Multimodal Cognitive Intervention for Adults Aging With HIV: A Focus Group Study. *J Assoc Nurses AIDS Care.* 2017 Sep-Oct;28(5):685-697.	Having the same dataset published in previous studies
11	Vance D, Fazeli P, Shacka J, Nicholson W, McKie P, Raper J, Azuero A, Wadley V, Ball K. Testing a Computerized Cognitive Training Protocol in Adults Aging With HIV-Associated Neurocognitive Disorders: Randomized Controlled Trial Rationale and Protocol. *JMIR Res Protoc.* 2017 Apr 26;6(4): e68.	Having the same dataset published in previous studies
12	Vance DE, Jensen M, Tende F, Raper JL, Morrison S, Fazeli PL. Individualized-Targeted Computerized Cognitive Training to Treat HIV-Associated Neurocognitive Disorder: An Interim Descriptive Analysis. *J Assoc Nurses AIDS Care*. 2018 Jul-Aug;29(4):604-611.	Not being RCT design
	Weber, E., Woods, S. P., Kellogg, E., Grant, I., Basso, M. R., & HIV Neurobehavioral Research Program (HNRP) Group (2012). Self-generation enhances verbal recall in individuals infected with HIV. Journal of the International Neuropsychological Society: JINS, 18(1), 128–133.	Not having computerized cognitive training
	Woods, S. P., Morgan, E. E., Loft, S., Matchanova, A., Verduzco, M., & Cushman, C. (2021). Enhancing cue salience improves aspects of naturalistic time-based prospective memory in older adults with HIV	Unpublished

© 2022 Wei J et al. *JAMA Network Open*.

	disease. Neuropsychology, 35(1), 111–122.	
	Woods, S. P., Morgan, E. E., Loft, S., Matchanova, A., Verduzco, M., & Cushman, C. (2020). Supporting strategic processes can improve time-based prospective memory in the laboratory among older adults with HIV disease. Neuropsychology, 34(3), 249–263.	Not a standard intervention study

© 2022 Wei J et al. *JAMA Network Open*.

eTable 4. Categorization of Outcome Measures

	Study name	Categorization of outcome measures
1	Chang et.al. (2016), United States	Digit span (A/WM); Spacial span (A/WM)
2	Cody et.al. (2019), United States	Digit copy test (MS); Letter comparison test (SIP); UFOV (A/WM)
3	Ezeamama et.al. (2020), Uganda	Color trails (SIP); Finger tapping (MS); Grooved pegboard test (Dominant hand) (SIP); Symbol digit modality (SIP); Timed gait (MS); Verbal fluency (V/L); WHO UCLA verbal learning test Delayed recall and recognition (M); WHO UCLA verbal learning test Delayed recall of first list (M); WHO UCLA verbal learning test Interference list recalls (M); WHO UCLA verbal learning test Post interference list recall (M); WHO UCLA verbal learning test Sum of immediate recalls (M)
4	Fazeli et.al. (2017), United States	Digit symbol substitution (SIP); Dimensional card change (A/E); Flanker computed (A/WM); Letter and pattern comparison (SIP); List sorting raw (A/WM); Oral reading recognition (V/L); Pattern comparison raw (SIP); Picture sequence memory (M); Picture vocabulary computed (V/L); UFOV 1 (SIP); UFOV 2 (A/WM); UFOV 3 (A/WM); UFOV risk (SIP); UFOV total (SIP)
5	Livelli et.al. (2015), Italy	Trail making test part A (SIP); Stroop color test-time (SIP); Rey auditory verbal learning test immediate recall (M); Rey auditory verbal learning test delayed recall (M); Rey-osterrieth complex figure delayed recall (M); Tower of London simplified version (A/E); Stroop color test-errors (A/E); Trail making test part B (A/E); Frontal assessment battery (A/E); Rey-osterrieth complex figure copy (A/E); Phonemic fluency (V/L); Verbal span (V/L); Corsi's block-Tapping test (A/WM); Digit span (A/WM); Trail making test part BA (A/WM); Grooved pegboard test dominant and non-dominant hands (MS); IADL test (DF)
6	Ownby et.al. (2016), United States	Digit span backward (A/WM); Digit span forward (A/WM); Digit span scaled score (A/WM); Digit span sequencing (A/WM); HVLT-R delayed recall (M); HVLT-R recognition (M); HVLT-R total (M); Pegs dominant hand (MS); Pegs nondominant hand (MS); Trails A (SIP); Trails B (A/E)
7	Pope et.al. (2018), United States	STISIM driving simulator (A/E)
8	Towe et.al. (2017), United States	Working memory (A/WM)
9	Towe et.al. (2020), United States	Paced auditory serial addition task-50 (A/WM); WAIS-Ⅳ Digit span subtest (A/WM); WAIS-Ⅳ Letter (A/WM);Trail making test part A (SIP); WAIS-ⅣCoding subtest (SIP); Stroop color and word test color naming score (SIP); HVLT-revised (M); BVMT-

© 2022 Wei J et al. *JAMA Network Open*.

		revised (M); Stroop color and word test interference score (A/E); Trail making test part B (A/E); WCST (A/E); FAS letter fluency (V/L); Category fluency (V/L); Grooved pegboard test dominant and non-dominant hand (MS)
10	Vance et.al. (2012), United States	Finger tapping test (MS); UFOV® test (A/WM); Wisconsin card sort test (A/E); TIADL test (DF)
11	Vance et.al. (2021a), United States	Stroop Color Naming Test, Trails A (SIP); Paced Auditory Serial Attention Test (A/WM); Stroop Interference, Trails B (A/E); Benton Visual Retention Test—Revised (M); Benton Visual Retention Test Delayed—Revised (M); WAIS IV Block Design (S-P); Hopkins Verbal Learning Test—Revised (M); Hopkins Verbal Learning Test Delayed—Revised (M)
12	Vance et.al. (2021b), United States	Stroop Color Naming Test, Trails A (SIP); Paced Auditory Serial Attention Test (A/WM); Stroop Interference, Trails B (A/E); Benton Visual Retention Test—Revised (M); Benton Visual Retention Test Delayed—Revised (M); WAIS IV Block Design (S-P); Hopkins Verbal Learning Test—Revised (M); Hopkins Verbal Learning Test Delayed—Revised (M)

A/E = Abstraction/executive; A/WM = Attention/working memory; M = Memory; MS = Motor skill; SIP = Speed of information processing; S-P = Sensory-perceptual; V/L = Verbal/language; DF = Daily function

© 2022 Wei J et al. *JAMA Network Open*.

eTable 5. Study Quality of Evidence According to GRADE Guidelines

CCT for HAND

Bibliography:

Outcomes	**No of Participants (studies)**<br>Follow up	**Quality of the evidence**<br>(GRADE)	**Relative effect (95% CI)**	**Anticipated absolute effects**	
				**Risk with Control**	**Risk difference with CCT** (95% CI)
**Abstraction/Executive**	240<br>(6 studies)<br>3-24 weeks	⊕⊕⊕⊝<br>**MODERATE**[1,2,3]<br>due to risk of bias, imprecision, large effect		See comment	See comment
**Attention/Working memory**	376<br>(9 studies)<br>3-24 weeks	⊕⊕⊕⊝<br>**MODERATE**[1,2,3]<br>due to risk of bias, imprecision, large effect			The mean attention/working memory in the intervention groups was<br>**0.62 standard deviations higher**<br>(0.33 to 0.91 higher)
**Memory**	245<br>(5 studies)<br>3-24 weeks	⊕⊕⊕⊝<br>**MODERATE**[1,3]<br>due to risk of bias, imprecision, large effect			The mean memory in the intervention groups was<br>**0.59 standard deviations higher**<br>(0.20 to 0.97 higher)
**Motor skill**	229<br>(5 studies)<br>3-10 weeks	⊕⊕⊕⊝<br>**MODERATE**[1,2,3]<br>due to risk of bias, imprecision, large effect			The mean motor skill in the intervention groups was<br>**0.50 standard deviations higher**<br>(0.24 to 0.77 higher)
**Speed of information processing**	246<br>(5 studies)<br>3-12 weeks	⊕⊕⊕⊝<br>**MODERATE**[1,2,3]<br>due to risk of bias, imprecision, large effect			The mean speed of information processing in the intervention groups was<br>**0.65 standard deviations higher**<br>(0.37 to 0.94 higher)
**Sensory-perceptual**	88<br>(1 study)<br>12 weeks	⊕⊝⊝⊝<br>**VERY LOW**[1,2,4]<br>due to risk of bias, inconsistency, imprecision, publication bias			The mean sensory-perceptual in the intervention groups was<br>**0.06 standard deviations higher**<br>(0.36 lower to 0.48 higher)

© 2022 Wei J et al. *JAMA Network Open*.

**Verbal/Language**	204<br>(4 studies)<br>4-24 weeks	⊕⊝⊝⊝<br>**VERY LOW**[1,2,3,5]<br>due to risk of bias, inconsistency, imprecision		The mean verbal/language in the intervention groups was<br>**0.46 standard deviations higher**<br>(0.07 lower to 0.99 higher)
**Daily function**	187<br>(3 studies)<br>5-24 weeks	⊕⊕⊕⊝<br>**MODERATE**[1,2,3]<br>due to risk of bias, imprecision, large effect		The mean daily function in the intervention groups was<br>**0.44 standard deviations higher**<br>(0.02 to 0.86 higher)

*The basis for the **assumed risk** (eg. the median control group risk across studies) is provided in footnotes. The **corresponding risk** (and its 95% confidence interval) is based on the assumed risk in the comparison group and the **relative effect** of the intervention (and its 95% CI).

CI: Confidence interval;

GRADE Working Group grades of evidence

High quality: Further research is very unlikely to change our confidence in the estimate of effect.

Moderate quality: Further research is likely to have an important impact on our confidence in the estimate of effect and may change the estimate.

Low quality: Further research is very likely to have an important impact on our confidence in the estimate of effect and is likely to change the estimate.

Very low quality: We are very uncertain about the estimate.

[1] Random sequence generation, allocation concealment, or blinding of outcome assessors were poorly described in 50% or more of included studies.

[2] Small sample size

[3] No explanation was provided

[4] Only one study, cannot be assessed.

[5] Large confidence intervals

© 2022 Wei J et al. *JAMA Network Open*.

eTable 6. Full Results of Sensitivity Analyses

Age

Outcomes	Age	SMD	95% CI	Z	*p*	*Q*	*p*
Abstraction/Executive				4.030	*<0.001*	2.373	*0.123*
	young(n=1)	1.137	0.390-1.884	2.984	*0.003*		
	old (n=5)	0.500	0.186-0.814	3.117	*0.002*		
Attention/Working memory				3.983	*<0.001*	0.699	*0.403*
	young(n=2)	0.976	-0.036-1.987	1.890	*0.059*		
	old (n=7)	0.527	0.241-0.814	3.605	*<0.001*		
Daily function				2.480	*0.013*	*3.170*	*0.075*
	young(n=1)	0.982	0.248-1.715	2.623	*0.009*		
	old (n=2)	0.255	-0.065-0.575	1.562	*0.118*		
Memory				4.167	*<0.001*	5.747	0.017
	young(n=1)	1.446	0.667-2.224	3.641	*<0.001*		
	old (n=4)	0.437	0.164-0.710	3.138	*0.002*		
Motor skill				3.739	*<0.001*	2.421	0.120
	young(n=1)	0.153	-0.362-0.688	0.582	0.561		
	old (n=4)	0.630	0.322-0.938	4.008	*<0.001*		
Speed of information processing	NA						

© 2022 Wei J et al. *JAMA Network Open*.

Proportion of women(%)

Outcomes	Sex	SMD	95% CI	Z	*p*	*Q*	*p*
Abstraction/Executive				3.070	*0.002*	0.002	*0.965*
	<50(n=4)	0.584	0.143-1.025	2.594	*0.009*		
	⩾50(n=2)	0.566	-0.110-1.241	1.642	*0.101*		
Attention/Working memory				4.125	*<0.001*	3.226	0.072
	<50(n=7)	0.738	0.411-1.066	4.424	*<0.001*		
	⩾50(n=2)	0.202	-0.282-0.687	0.818	*0.413*		
Daily function	NA						
Memory				2.564	*0.010*	0.531	*0.466*
	<50(n=3)	0.775	0.031-1.520	2.040	*0.041*		
	⩾50(n=2)	0.441	-0.063-0.945	1.716	*0.086*		
Motor skill				3.448	*0.001*	0.022	0.882
	<50(n=3)	0.496	-0.001-0.993	1.957	0.050		

© 2022 Wei J et al. *JAMA Network Open*.

	⩾50(n=2)	0.543	0.169-0.918	2.843	0.004		
Speed of information processing				4.974	*<0.001*	2.640	0.104
	<50(n=2)	0.926	0.510-1.343	4.357	*<0.001*		
	⩾50(n=3)	0.486	0.157-0.815	2.898	*0.004*		

Years of Education (years)

Outcomes	Education	SMD	95% CI	Z	p	Q	p
Abstraction/Executive				3.714	<0.001	1.273	0.259
	⩽12(n=3)	0.762	0.328-1.196	3.443	0.001		
	>12(n=3)	0.405	-0.038-0.848	1.793	0.073		
Attention/Working memory				3.927	<0.001	0.279	0.597
	⩽12(n=4)	0.727	0.246-1.208	2.960	0.003		
	>12(n=5)	0.556	0.142-0.969	2.634	0.008		
Daily function				1.914	0.056	0.043	0.836

© 2022 Wei J et al. *JAMA Network Open*.

	≤12(n=2)	0.520	-0.251-1.290	1.322	0.186		
	>12(n=1)	0.418	-0.167-1.002	1.399	0.162		
Memory				2.850	0.004	2.011	0.156
	≤12(n=4)	0.694	0.265-1.124	3.168	0.002		
	>12(n=1)	0.110	-0.573-0.793	0.316	0.752		
Motor skill				3.739	<0.001	2.856	0.091
	≤12(n=3)	0.344	0.021-0.667	2.089	0.037		
	>12(n=2)	0.829	0.369-1.289	3.532	<0.001		
Speed of information processing				3.697	<0.001	0.005	0.945
	≤12(n=3)	0.642	0.158-1.126	2.601	0.009		
	>12(n=2)	0.666	0.170-1.163	2.629	0.009		

$CD4^{+}T$ Cell Counts (cells/ul)

Outcomes	CD4	SMD	95% CI	Z	*p*	*Q*	*p*

© 2022 Wei J et al. *JAMA Network Open*.

Abstraction/Executive				3.878	<0.001	2.233	*0.135*
	<500(n=1)	0.181	-0.399-0.761	0.613	*0.540*		
	⩾500(n=5)	0.689	0.361-1.018	4.110	<0.001		
Attention/Working memory				4.166	*<0.001*	4.776	*0.029*
	<500(n=2)	0.198	-0.185-0.580	1.013	*0.311*		
	⩾500(n=7)	0.755	0.433-1.078	4.594	*<0.001*		
Daily function				2.127	*0.033*	1.904	*0.168*
	<500(n=2)	0.654	0.109-1.200	2.350	*0.019*		
	⩾500(n=1)	0.185	-0.197-0.567	0.951	*0.342*		
Memory				3.048	*0.002*	0.779	*0.377*
	<500(n=1)	0.297	-0.213-0.807	1.140	*0.254*		
	⩾500(n=5)	0.585	0.198-0.972	2.962	*0.003*		
Motor skill				3.150	0.002	2.137	0.144
	<500(n=1)	0.855	0.251-1.460	2.774	0.006		

© 2022 Wei J et al. *JAMA Network Open*.

	⩾500(n=3)	0.344	0.021-0.667	2.089	0.037		
Speed of information processing				3.276	*0.001*	1.657	0.198
	<500(n=1)	0.209	-0.299-0.718	0.807	0.420		
	⩾500(n=4)	0.616	0.264-0.968	3.426	*0.001*		

HIV suppression rate (%)

Outcomes	HIV suppression rate	SMD	95% CI	Z	*p*	*Q*	*p*
Abstraction/Executive				3.035	*0.002*	3.391	*0.066*
	<100(n=4)	0.357	-0.007-0.720	1.924	*0.054*		
	⩾100(n=1)	1.137	0.390-1.884	2.984	*0.003*		
Attention/Working memory				5.494	*<0.001*	*4.521*	*0.033*
	<100(n=6)	0.631	0.355-0.907	4.484	*<0.001*		
	⩾100(n=1)	1.537	0.749-2.326	3.821	*<0.001*		
Daily function				2.730	*0.006*	1.390	*0.238*

© 2022 Wei J et al. *JAMA Network Open*.

	<100(n=1)	0.418	-0.167-1.002	1.399	*0.162*		
	⩾100(n=1)	0.982	0.248-1.715	2.623	*0.009*		
Memory				3.977	*<0.001*	4.755	*0.029*
	<100(n=3)	0.493	0.136-0.850	2.705	*0.007*		
	⩾100(n=1)	1.446	0.667-2.224	3.641	*<0.001*		
Motor skill	NA						
Speed of information processing	NA						

Session

Outcomes	session	SMD	95% CI	Z	*p*	*Q*	*p*
Abstraction/Executive				3.140	*0.002*	2.090	*0.148*
	⩾22(n=3)	0.465	-0.057-0.987	1.747	*0.081*		
	<22(n=1)	1.137	0.390-1.884	2.984	*0.003*		
Attention/Working memory				3.698	*<0.001*	1.341	*0.247*
	⩾22(n=4)	0.509	-0.002-1.021	1.950	*0.051*		

© 2022 Wei J et al. *JAMA Network Open*.

	<22(n=3)	0.958	0.397-1.519	3.348	*0.001*		
Daily function	NA						
Memory				2.098	*0.036*	2.236	0.135
	⩾22(n=2)	0.230	-0.365-0.826	0.758	0.449		
	<22(n=2)	0.978	0.200-1.756	2.463	*0.014*		
Motor skill				2.809	0.005	1.445	0.229
	⩾22(n=2)	0.751	0.138-1.363	2.403	0.016		
	<22(n=2)	0.323	-0.012-0.658	1.887	0.059		
Speed of information processing				3.008	*0.003*	0.831	0.362
	⩾22(n=3)	0.641	0.182-1.101	2.737	0.006		
<	<22(n=1)	0.346	-0.094-0.785	1.545	*0.122*		

Session hours (minutes)

Outcomes	Session hours	SMD	95% CI	Z	*p*	*Q*	*p*
Abstraction/Executive				2.130	*0.033*	0.213	*0.644*

© 2022 Wei J et al. *JAMA Network Open*.

	<60(n=2)	0.679	-0.396-1.754	1.239	*0.216*		
	⩾60(n=3)	0.405	-0.038-0.848	1.793	*0.073*		
Attention/Working memory				4.325	*<0.001*	9.410	*0.002*
	<60 (n=3)	1.150	0.712-1.589	5.138	*<0.001*		
	⩾60 (n=3)	0.249	-0.123-0.622	1.312	*0.189*		
Daily function				2.480	*0.013*	*3.170*	*0.075*
	<60 (n=1)	0.982	0.248-1.715	2.623	*0.009*		
	⩾60 (n=2)	0.255	-0.065-0.575	1.562	*0.118*		
Memory				2.703	*0.007*	3.016	0.082
	<60 (n=3)	0.882	0.341-1.423	3.198	0.001		
	⩾60 (n=1)	0.110	0.161-1.009	0.316	*0.752*		
Motor skill				4.008	*<0.001*	1.305	0.253
	<60 (n=2)	0.468	0.053-0.882	2.212	0.027		

© 2022 Wei J et al. *JAMA Network Open*.

	⩾60 (n=2)	0.829	0.369-1.289	3.532	*<0.001*		
Speed of information processing				3.008	*0.003*	0.847	0.358
	<60 (n=2)	0.363	-0.049-0.776	1.727	0.084		
	⩾60 (n=2)	0.666	0.170-1.163	2.629	*0.009*		

Frequency (times/week)

Outcomes	frequency	SMD	95% CI	Z	*p*	*Q*	*p*
Abstraction/Executive	NA						
Attention/Working memory				3.703	*<0.001*	0.375	*0.540*
	<3(n=2)	0.578	-0.586-1.741	0.973	*0.330*		
	⩾3(n=2)	0.977	0.449-1.505	3.625	*<0.001*		
Daily function	NA						
Memory				2.979	*0.003*	0.002	*0.961*
	<3(n=1)	0.641	0.194-1.088	2.813	*0.005*		
	⩾3(n=1)	0.609	-0.605-1.822	0.983	*0.326*		

© 2022 Wei J et al. *JAMA Network Open*.

Motor skill				3.015	*0.003*	0.017	*0.895*
	<3(n=2)	0.543	0.169-0.918	2.843	*0.004*		
	⩾3(n=1)	0.629	-0.587-.844	1.014	*0.311*		
Speed of information processing				2.299	*0.021*	0.001	0.970
	<3(n=2)	0.519	0.047-0.992	2.154	0.031		
	⩾3(n=1)	0.495	-0.710-1.699	0.805			

Total training hours (hours)

Outcomes	hours	SMD	95% CI	Z	*p*	*Q*	*p*
Abstraction/Executive	NA						
Attention/Working memory				3.635	*<0.001*	1.854	*0.173*
	<12(n=4)	0.478	-0.018-0.974	1.890	*0.059*		
	⩾12(n=1)	1.011	0.427-1.595	3.391	*0.001*		
Daily function	NA						
Memory	NA						

© 2022 Wei J et al. *JAMA Network Open*.

Motor skill	NA						
Speed of information processing	NA						

Time since training (weeks)

Outcomes	Follow-up	SMD	95% CI	Z	*p*	*Q*	*p*
Abstraction/Executive				3.842	*<0.001*	5.326	*0.021*
	<10(n=3)	0.183	-0.232-0.598	0.865	*0.387*		
	⩾10(n=2)	0.841	0.466-1.216	4.397	*<0.001*		
Attention/Working memory				3.956	*<0.001*	1.040	*0.308*
	<10(n=5)	0.487	0.121-0.854	2.605	*0.009*		
	⩾10(n=4)	0.812	0.306-1.318	3.147	*0.002*		
Daily function				1.914	*0.056*	0.043	*0.836*
	<10(n=1)	0.418	-0.167-1.002	1.399	*0.162*		
	⩾10(n=2)	0.520	-0.251-1.290	1.322	*0.186*		
Memory				3.136	*0.002*	0.495	0.482

© 2022 Wei J et al. *JAMA Network Open*.

	<10(n=6)	0.454	0.136-0.771	2.803	*0.005*		
	⩾10(n=2)	0.851	-0.210-1.912	1.573	*0.116*		
Motor skill				3.739	*<0.001*	2.421	0.120
	<10(n=4)	0.630	0.322-0.938	4.008	*<0.001*		
	⩾10(n=1)	0.153	-0.362-0.668	0.582	0.561		
Speed of information processing				4.974	*<0.001*	3.200	0.074
	<10(n=4)	0.487	0.170-0.804	3.008	0.003		
	⩾10(n=1)	0.985	0.541-1.429	4.347	*<0.001*		

Type of participants

Outcomes	Type of participants	SMD	95% CI	Z	*p*	*Q*	*p*
Abstraction/Executive				3.714	*<0.001*	1.272	*0.259*
	Cognitive normal(n=3)	0.405	-0.038-0848	1.793	*0.073*		
	Cognitive impairment(n=3)	0.762	0.328-1.196	3.443	*0.001*		
Attention/Working memory				3.891	*<0.001*	0.625	*0.429*

© 2022 Wei J et al. *JAMA Network Open*.

	Cognitive normal(n=6)	0.540	0.219-0.862	3.295	*<0.001*		
	Cognitive impairment(n=3)	0.880	0.102-1.658	2.216	*0.027*		
Daily function				1.914	*0.056*	0.043	*0.836*
	Cognitive normal(n=1)	0.418	-0.167-1.002	1.399	*0.162*		
	Cognitive impairment(n=2)	0.520	-0.011-0.921	1.322	*0.186*		
Memory				2.564	*0.010*	0.531	0.466
	Cognitive normal(n=2)	0.441	-0.063-0.945	1.716	*0.086*		
	Cognitive impairment(n=3)	0.775	0.031-1.520	2.040	*0.041*		
Motor skill				3.390	*0.001*	0.035	0.852
	Cognitive normal(n=4)	0.510	0.201-0.818	3.240	*0.001*		
	Cognitive impairment(n=1)	0.629	-0.587-1.844	1.014	0.311		
Speed of information processing				4.972	*<0.001*	2.640	0.104
	Cognitive normal(n=3)	0.486	0.157-0.815	2.898	*0.004*		
	Cognitive impairment(n=2)	0.926	0.510-1.343	4.357	*<0.001*		

© 2022 Wei J et al. *JAMA Network Open*.

eFigure. Risk of Bias Summary

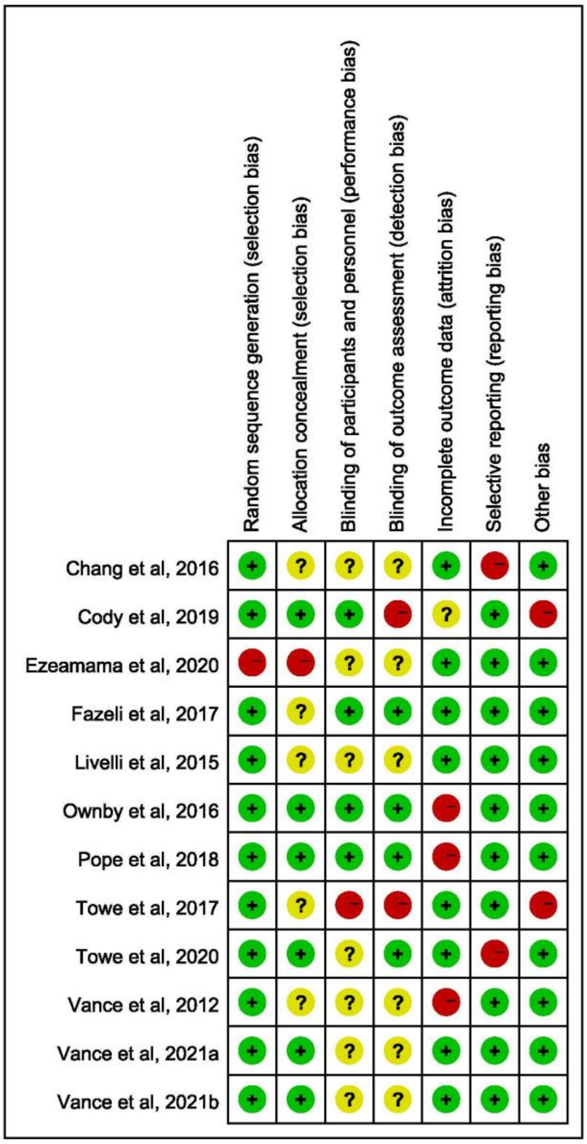

© 2022 Wei J et al. *JAMA Network Open*.
